# Quality of life status determinants in hypertrophic cardiomyopathy as evaluated by the Kansas City Cardiomyopathy Questionnaire

**DOI:** 10.1186/s12955-020-01604-9

**Published:** 2020-10-30

**Authors:** Razvan Capota, Sebastian Militaru, Alin Alexandru Ionescu, Monica Rosca, Cristian Baicus, Bogdan Alexandru Popescu, Ruxandra Jurcut

**Affiliations:** 1 Expert Center for Rare Genetic Cardiovascular Diseases, Emergency Institute for Cardiovascular Diseases “Prof.Dr.C.C.Iliescu”, Sos. Fundeni 258, 022322 Bucharest, Romania; 2 Colentina Clinical Hospital, Bucharest, Romania; 3 University of Medicine and Pharmacy “Carol Davila”, Bucharest, Romania

**Keywords:** Hypertrophic cardiomyopathy, Quality of life, Heart failure, Kansas city cardiomyopathy questionnaire

## Abstract

**Purpose:**

The present study evaluated how heart failure (HF) negatively impacts health-related quality of life (HRQoL) in hypertrophic cardiomyopathy (HCM) patients and explored the major clinical determinants associated with HRQoL impairment in this population.

**Methods:**

This was a cross-sectional single-center study of health-related HRQoL that included 91 consecutive patients with HCM. Evaluation was performed based on a comprehensive protocol that included the recommended diagnostic studies, as well as administration of the translated validated version of the Kansas City Cardiomyopathy Questionnaire (KCCQ) (CV Outcomes Inc) as a health status measure.

**Results:**

The cohort included 52 (57%) males, median age 58 (20–85) years. The median global KCCQ score was 67 (12.5–100) corresponding to a moderate impairment in HRQoL. There was an inverse correlation between the median global KCCQ score and NYHA class (Kendall’s tau b coefficient r − 0.33, p = 0.001). Patients with pulmonary hypertension (PHT), defined as resting pulmonary artery systolic pressure of ≥ 45 mmHg, presented a significantly worse HRQoL as compared to those without PHT (median KCCQ score 56.2 vs 77.5, p = 0.013). The KCCQ score mildly correlated with age (r − 0.18, p = 0.014), history of syncope (r − 0.18, p = 0.045), estimated glomerular filtration rate (eGFR) (r 0.31, p < 0.001), plasmatic creatinine (r − 0.18, p = 0.017) and urea levels (r − 0.27, p < 0.001), left ventricular (LV) end-systolic dimensions (r − 0.18, p = 0.014), maximal provoked intraventricular gradient (r 0.20, p = 0.039), LV ejection fraction (r 0.15, p = 0.04), average E/e′ (r − 0.16, p = 0.039), pulmonary acceleration time (r 0.21, p = 0.007), pulmonary artery systolic pressure (r − 0.20, p = 0.016). In ordinal regression, the independent predictors of HRQoL were NYHA class and eGFR.

**Conclusions:**

Patients with HCM and HF present a moderate degree of alteration in HRQoL. This is especially true for patients with PHT and more severe functional impairment. Renal failure and NYHA class are potential markers of HRQoL in clinical practice.

## Background

Health related quality of life (HRQoL) in patients with hypertrophic cardiomyopathy (HCM) is often overlooked in routine clinical practice.

HCM is a genetically determined heart condition characterized by left ventricular hypertrophy (LVH) of various morphologies most often caused by mutations in sarcomere genes, which encode different components of the myocardial contractile apparatus [1]. According to recent epidemiological data, HCM is the most common inherited cardiovascular disorder, affecting up to 1 in 500 individuals worldwide [2].

The most frequent clinical manifestations in HCM patients are related to heart failure (HF), with exertional dyspnea occurring in over 90% of symptomatic patients [3, 4]. Moreover, most of these symptomatic patients display New York Heart Association (NYHA) class II or greater dyspnea at presentation [3] This may be related to different mechanisms of disease such as diastolic dysfunction, left ventricular outflow tract obstruction, mitral regurgitation, myocardial ischemia, atrial fibrillation and in a small percent of patients, progression to a “burnt-out” phase marked by the presence of systolic dysfunction [3, 4]. The relative contribution of each of these mechanisms may change with time and stage of disease.

While frequently overlooked in routine clinical practice, HRQoL is a global measure of disease-related symptom burden and an important clinical objective. We need to acknowledge the fact that all therapeutical interventions ideally aim to improve both symptoms (translated into HRQoL) and prognosis. While there is abundant data regarding interventions and strategies to improve long-term prognosis, very little information is currently available on HRQoL and its determinant factors in HCM patients. In a tertiary center study, HCM patients had deteriorated HRQoL compared to the general population [5]. Interestingly, several studies showed no impairment of HRQoL in screening diagnosed cardiomyopathies carriers compared to the general population [6, 7], suggesting that focus should be on symptomatic patients. To our knowledge, this is the first study looking at HRQoL determinants in symptomatic HCM patients using the KCCQ as a health status measure.

The primary aim of the study was to evaluate the level of HRQoL in patients with HCM and HF from a national referral center. The secondary aims included the identification of predictors as well as potential differences in HRQoL between different subpopulations.

## Methods

### Study population

We performed a cross-sectional single-center study including all consecutive HCM patients diagnosed according to current ESC guidelines who were referred for evaluation between June 2016 and January 2017 at the Expert Center for Rare Genetic Cardiovascular Diseases at “Prof. Dr. C. C. Iliescu” Institute for Cardiovascular Diseases. To be eligible for the study, patients had to present: (1) the sarcomeric form of HCM defined by a wall thickness ≥ 15 mm in any patient and/or ≥ 13 mm in family members of a confirmed HCM index case in one or more LV myocardial segments as measured by echocardiography that is not solely explained by abnormal loading conditions and (2) signs and/or symptoms of HF.

Patients were excluded if other causes of LV hypertrophy (LVH) were strongly suspected or already confirmed (so called phenocopies) which included: inborn errors of metabolism (glycogen/lysosomal storage diseases), drug-induced LVH, mitochondrial disease, malformation syndromes or amyloidosis. Patient medical records were reviewed and the following data were included: age, sex, type of HCM—obstructive (HOCM) or non-obstructive (NHOCM), clinical examination findings, comorbid conditions, laboratory, electrocardiographic and imaging results.

Patients participating in this study provided written and oral informed consent permitting use of patient medical information for research.

### Quality of life evaluation

All patients were administered the translated validated version of the Kansas City Cardiomyopathy Questionnaire (KCCQ) (CV Outcomes Inc) as a health status measure [5, 8]. KCCQ is a patient-reported multidimensional instrument used in numerous studies since its introduction. It is useful for estimating the perceived burden of heart failure symptoms [5, 8]. The KCCQ is a self-administered questionnaire with scores ranging from 0 to 100, with higher values representing better HRQoL. The summary KCCQ score represents the mean score of the questionnaire’s different assessment domains: physical limitation (range 0–100), symptom frequency (range 0–100), quality of life (range 0–100) and social limitation (range 0–100), which we also analyzed separately.

### Definition of variables

Patients were considered to present HOCM if an intraventricular gradient ≥ 30 mmHg in the resting state or after provocation maneuvers (either Valsalva maneuver or sublingual nitroglycerine) was identified and NHOCM if they did not present such an intraventricular gradient. Estimated glomerular filtration rate (eGFR) was determined using the online version of the Modification of Diet in Renal Disease (MDRD) Study equation based on creatinine and patient characteristics. Transthoracic echocardiography image acquisition and measurements were performed according to current European Association of Cardiovascular Imaging (EACVI) guidelines and recommendations [9]. LV ejection fraction (LVEF) and volumetric assessment was performed using the Simpson’s biplane method. Pulmonary hypertension (PHT) was defined as resting pulmonary artery systolic pressure of ≥ 45 mmHg based on the maximum tricuspid regurgitation jet velocity measured by transthoracic echocardiography (TTE); pulmonary acceleration time (PAT) was also recorded as part of the PHT evaluation. History of atrial fibrillation was defined as at least one documented episode of atrial fibrillation having minimum 30 s duration on ECG or Holter monitoring.

### Statistical analysis

Patient characteristics for the entire group were summarized using the median (min, max) for continuous and ordinal variables and counts (percentage) for categorical variables. Normality of data was tested using the Shapiro–Wilk test.

Data analysis was performed using non-parametric tests. Comparisons between 2 groups were performed using the Mann–Whitney *U* test for continuous and ordinal variables and Fisher’s exact test for categorical variables.

The Jonckheere–Tepstra test followed by post-hoc pairwise analysis if the test was significant at the 0.05 level was used when considering more than two groups for continuous and ordinal variables. Non-parametric correlation analysis using Kendall’s tau-b correlation coefficient and ordinal regression analysis were conducted to identify variables that were associated with HRQoL. Variables with a significant Kendall’s tau-b correlation coefficient were initially considered in an ordinal regression model and reduced by backward elimination. A two-sided p value of less than 0.05 was considered statistically significant for all tests. IBM SPSS Statistics version 23 (SPSS, IBM, Armonk, New York) was used for data analysis.

### Sample size

For the estimation of the mean KCCQ score with a presumed standard deviation of 20 points, with an error of ± 5 points and 95% confidence, we calculated a sample size of 64 patients.

## Results

### Baseline characteristics

A total of 91 patients with HCM and HF were included. Baseline demographic and clinical characteristics data are summarized in Table 1. Participants were predominantly male (57%), with a median age of 58 (20–85) years. The NYHA class distribution was the following: 22 patients in NYHA I (24.2%), 49 patients in NYHA II (53.8%), 19 patients in NYHA III (20.9%), and 1 patient in NYHA IV (1.1%). Regarding phenotypic expression, 54% of patients presented obstructive HCM (HOCM) while 46% presented non-obstructive HCM (NHOCM). Median Brain Natriuretic Peptide (BNP) and LVEF values were 296 (10–3999) pg/ml and 60% (29–88%) respectively. Pulmonary hypertension estimation was possible in 70 patients (77%) using tricuspid regurgitation peak velocity, and 24 patients (34% of measured) presented with PHT.

**Table 1:** Baseline characteristics in the study group

Variable	Results
Age(y), median (min, max)	58 (20–85)
Age at diagnosis (y), median (min, max)	53 (9–85)
Gender, n (%)	
Male	52 (57%)
Female	39 (43%)
Phenotype, n (%)	
Obstructive	49 (54%)
Nonobstructive	42 (46%)
NYHA class, n (%)	
I	22 (24.2%)
II	49 (53.8%)
III	19 (20.9%)
IV	1 (1.1%)
Systemic hypertension, n (%)	52 (61.9%)
Diabetes, n (%)	12 (14.3%)
Chronic Kidney disease, n (%)	21 (25%)
ICD, n (%)	7 (8.1%)
Pacemaker, n (%)	10 (11.6%)
Pulmonary hypertension, n (% of measured)	24 (34%)
Rhythm n (%)	
Sinus rhythm	67 (73.6%)
Atrial fibrillation	9 (9.9%)
Other	15 (16.5%)
BNP (pg/ml), median (min,max)	296 (10–3999)
Coronary artery disease, n (%)	8 (12.1%)

*BNP* brain natriuretic peptide, *ICD* implantable cardiac defibrillator, *NYHA* New York Heart Association, *n* number, *y* years

### Quality of life evaluation and correlates

The global HRQoL reflected by the median KCCQ score was 67 (12.5–100) points corresponding to a moderate degree of impairment in HRQoL in our cohort. The symptom frequency assessment domain had the highest median score 75 (0–100) points while the lowest median score was 62.5 (12.5–100) points in the quality of life assessment domain (Table 2). Females presented a worse HRQoL with lower global KCCQ scores (median score 56.2 vs 78.1, p = 0.04) and a higher symptom burden (median score 64.5 vs 91.6, p = 0.006) as compared to male patients, though there were no differences between the two genders (females vs males) regarding physical limitation (median score 58.3 vs 75 p = 0.058), social limitation (median score 62.5 vs 79.1 p = 0.08) and quality of life assessment domains’ scores (median score 62.5 vs 62.5 p = 0.54).

**Table 2:** Kansas City Cardiomyopathy Questionnaire results summary for each of the assessment domains and global KCCQ score

Variable	Results, median (min, max)
Physical limitation	75 (0–100)
Symptom frequency	79 (20.8–100)
Quality of life	62.5 (12.5–100)
Social limitation	75 (0–100)
Global KCCQ	67 (12.5–100)

*KCCQ* Kansas City Cardiomyopathy Questionnaire

Patients in NYHA class I had a superior HRQoL with higher median KCCQ scores as compared to patients in NYHA class II, 90.6 versus 65.6 points (p = 0.002), as well as NYHA class III patients, 90.6 versus 57.2 points (p = 0.001), but there were no significant differences between patients presenting class II and III NYHA symptoms, 65.6 versus 57.2 points (p = 0.360) (Table 3). There were also significant differences between patients in NYHA class I and class III in all of the questionnaire’s assessment domains—the physical limitation domain, median 95.8 versus 50 points (p = 0.001), the symptom frequency domain, median 100 versus 64.5 points (p = 0.001), the quality of life domain, median 87.5 versus 50 points (p = 0.004) and the social limitation domain, median 100 versus 50 points (p = 0.003). In addition, class I NYHA and class II NYHA patients presented significant differences in the physical limitation domain, median 95.8 versus 64.5 points (p = 0.002), the quality of life domain, median 87.5 versus 50 points (p = 0.018) and the symptom frequency domain, median 100 versus 75 points (p = 0.001).

**Table 3:** Kansas City Cardiomyopathy Questionnaire results summary for each of the assessment domains and global KCCQ score according to NYHA functional class

Variable	NYHA I median (min, max)	NYHA II median (min, max)	NYHA III median (min, max)	P Jonckheere–Tepstra	Pairwise posttest comparissons
Physical limitation	95.8 (41.6–100)	64.5 (0–100)	50 (25–87.5)	0.001	1–2: p = 0.002 1–3: p = 0.001
Symptom frequency	100 (52–100)	75 (20.8–100)	64.5 (39.5–100)	0.001	1–2: p = 0.001 1–3: p = 0.001
Quality of life	87.5 (12.5–100)	50 (12.5–100)	50 (12.5–100)	0.002	1–3: p = 0.004 1–2: p = 0.018
Social limitation	100 (8.3–100)	75 (0–100)	50 (8.3–100)	0.002	1–3: p = 0.003
Global KCCQ	90.6 (35.9–100)	65.6 (19.4–100)	57.2 (30.7–88.5)	0.001	1–3: p = 0.001 1–2: p = 0.002

*NYHA* New York Heart Association, *KCCQ* Kansas City Cardiomyopathy Questionnaire

Patients with PHT had a significantly worse HRQoL as compared to patients without PHT, presenting lower median KCCQ scores, 56.2 versus 77.5 points (p = 0.013). They also presented significant differences in most of the questionnaire’s assessment domains: the physical limitation summary score, median 50 versus 75 points (p = 0.004), the quality of life summary score, median 50 versus 75 points (p = 0.026) and the social limitation summary score, median 58.3 versus 83.3 points (p = 0.033), while no significant differences were observed in the symptom frequency summary score, median 75 versus 81.2 points (p = 0.299). Patients with a permanent pacemaker presented lower scores in the symptom frequency summary score, median scores of 47.9 versus 81.2 points (p = 0.046), without any significant differences in global HRQoL, median KCCQ scores of 54.4 versus 69.2 points (p = 0.085), or any of the other questionnaire’s assessment domains. The study cohort included 7 patients with ICD (8.1%) and while these patients presented a trend towards a worse HRQoL with lower median KCCQ scores, the observed difference was not significant, 57.2 points versus 67 points, p = 0.67.

There were no significant differences in HRQoL between patients with HOCM and NHOCM, both groups presenting similar median KCCQ scores (76 points in the HOCM group versus 66.1 points in the NHOCM group, p = 0.29). There were also no significant differences observed between the two groups in the questionnaire’s assessment domains: the physical limitation summary score, median 75 versus 66.6 points (p = 0.60), the symptom frequency summary score, median 87.5 versus 76.3 points (p = 0.126), the quality of life summary score, median 75 versus 50 points (p = 0.125) and in the social limitation summary score, median 75 versus 70.8 points (p = 0.598).

The KCCQ score presented fair correlations with the eGFR (creatinine clearance), plasmatic urea level and NYHA functional class. The mean KCCQ score also presented weak but significant correlations with age, previous history of syncope, R wave amplitude in the aVL lead on resting ECG and various echocardiographic parameters: 2D-LV end-systolic dimensions, LVEF, average peak mitral inflow-to-mitral relaxation velocity ratio E/e’, pulmonary artery systolic pressure (PASP), pulmonary acceleration time (PAT) and maximal provoked intraventricular gradient (Table 4).

**Table 4:** Correlations between the global KCCQ score and different parameters

Variable	Global KCCQ Kendall’s tau-b correlation coefficient	Sig. (p value)
Age (y)	− 0.18	0.011
Chronic kidney disease	− 0.22	0.013
History of syncope	− 0.18	0.045
NYHA functional class	− 0.33	< 0.001
eGFR (mL/min/1.73 m2)	0.31	< 0.001
Serum urea (mg/dl)	− 0.27	< 0.001
Serum creatinine (mg/dl)	− 0.18	0.017
R wave amplitude in aVL lead (mm)	− 0.16	0.042
LVEF (%)	0.15	0.04
PASP (mmHg)	− 0.20	0.016
PAT (ms)	0.21	0.007
Average E/e′ ratio	− 0.16	0.039
Pulmonary hypertension	− 0.23	0.011
Max provoked intraventricular gradient (mmHg)	0.20	0.039
LV ESD (mm)	− 0.18	0.014
IVS thickness (mm)	− 0.11	0.109
LA antero-posterior diameter (mm)	0.05	0.499
BNP (pg/ml)	− 0.10	0.252

*LV ESD* left ventricular end-systolic dimension, *LVEF* left ventricular ejection fraction, *PASP* pulmonary artery systolic pressure, *PAT* pulmonary acceleration time, *E/e′ ratio* peak mitral inflow-to-mitral relaxation velocity ratio, *IVS* interventricular septum, *LA* left atrium, *BNP* brain natriuretic peptide

**Fig. 1:**
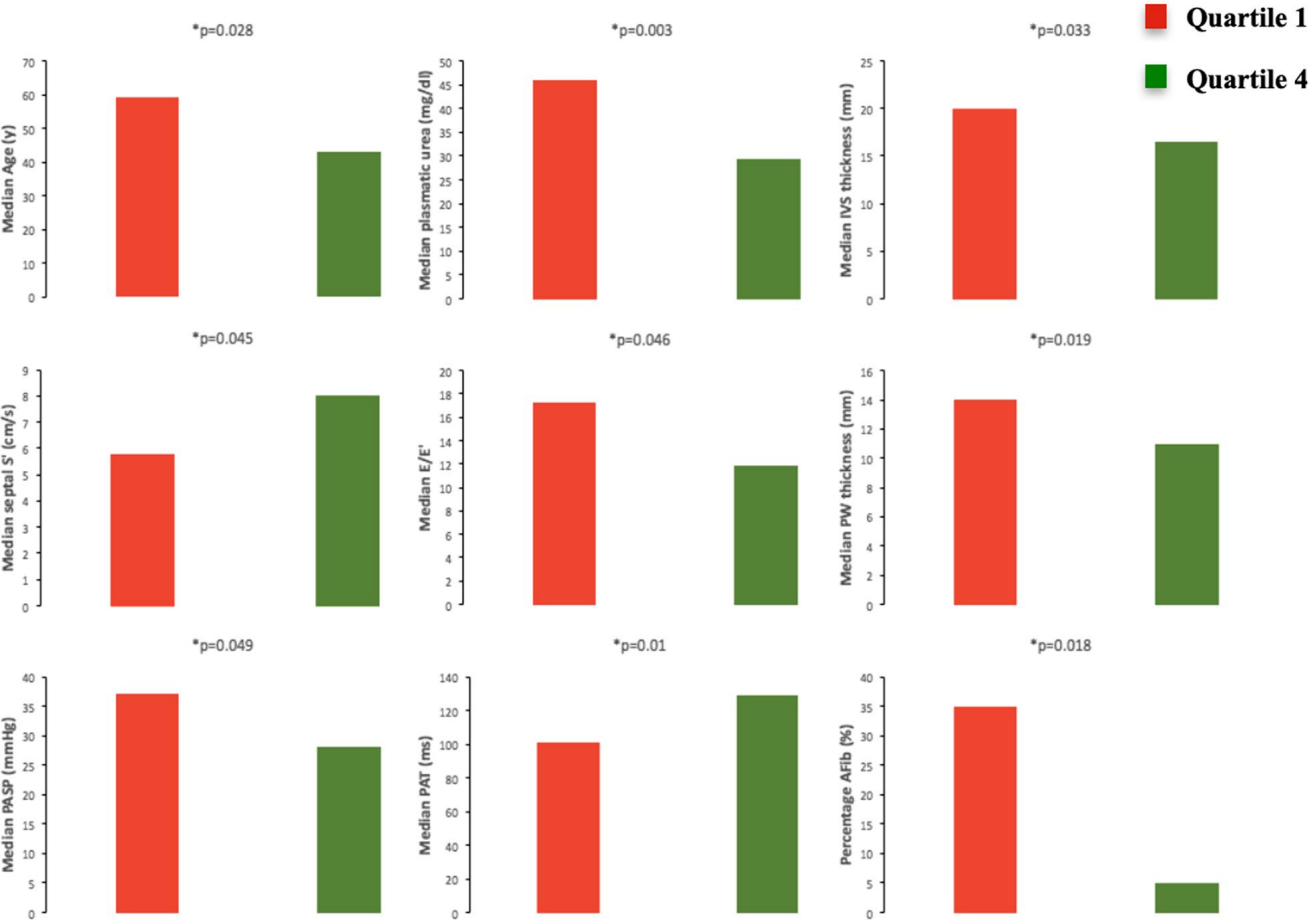
Comparisons between the first (Q1) and fourth (Q4) quartile of distribution of the KCCQ score regarding age (y), history of atrial fibrillation—AFib (%), interventricular septum—IVS thickness (mm), posterior wall—PW thickness (mm), E/E′ ratio—peak mitral inflow-to-mitral relaxation velocity ratio, pulmonary artery systolic pressure—PASP (mmHg), pulmonary acceleration time—PAT (ms) and septal longitudinal systolic velocities—septal S′ (cm/s)

We also evaluated the potential differences between patients with the highest HRQoL, defined as patients belonging to the fourth quartile of distribution of the KCCQ score, and patients with the worst HRQoL, defined as patients belonging to the first quartile of distribution of the KCCQ score (Fig. 1). Patients in the worst HRQoL group were older (median age 59 vs 43 years, p = 0.028), presented higher median plasmatic creatinine levels (1.08 vs 0.86 mg/dl, p = 0.044) and plasmatic urea levels (46 vs 29.5 mg/dl, p = 0.003), higher median interventricular septal (IVS) thickness (20 vs 16.5 mm, p = 0.033) and posterior wall thickness (14 vs 11 mm, p = 0.019), lower median septal s’ velocities (5.8 vs 8 cm/s p = 0.045), higher median E/e′ (17.3 vs 11.9, p = 0.046), lower median PAT values (101 vs 129 ms, p = 0.01) and higher median PASP (37 vs 28 mmHg p = 0.049). Patients with a low HRQoL level also presented an increased symptom burden with significantly more patients having class II and III NYHA dyspnea (14 vs 8 patients with class II NYHA symptoms, 5 versus 0 patients with class III NYHA symptoms, p = 0.001); they also demonstrated an increased frequency of atrial fibrillation, presenting a likelihood ratio of 6.1 (p = 0.013) of having had at least one documented episode of atrial fibrillation and an increased prevalence of PHT presenting a likelihood ratio of 7.9 (p = 0.005).

Ordinal regression analysis identified NYHA class and eGFR (creatinine clearance) as predictive factors for HRQoL quantified by means of the KCCQ score, with an adjusted R^2^ of 0.32, p < 0.001 (Table 5).

**Table 5:** Ordinal regression analysis identified NYHA class and eGFR (creatinine clearance) as predictive factors using the KCCQ score as the dependent variable

Model	Estimate	SE	Wald	Sig. (p value)	95.0% CI for B	
					Lower bound	Upper bound
NYHA class	− 1.11	0.305	13.43	< 0.001	− 1.713	− 0.519
eGFR (ml/min/1.73 m^2^)	0.035	0.010	13.50	< 0.001	0.016	0.054

*eGFR* estimated glomerular filtration rate, *KCCQ* Kansas City Cardiomyopathy Questionnaire, *NYHA* New York Heart Association

## Discussion

The present study demonstrated that, even in the modern therapeutic era, patients with HCM and HF report a moderate degree of impairment in HRQoL.

Patient reported outcome measures (PROM) are an important tool for understanding patients perception of their functional well-being and health status. The KCCQ is a valid, reliable and responsive health status measure for patients with HF and was demonstrated by many studies to be a clinically meaningful outcome in cardiovascular research, patient management and quality assessment [10]. Moreover, the International Consortium for Health Outcomes Measurement recently published a statement [11] including KCCQ as one of the most commonly used PROM in heart failure, well validated and widely studied.

Our findings are in agreement with previous studies that demonstrated a significantly worse HRQoL in the HCM patient population as compared to sex and age matched patients from the general population, including in specific patient populations such as implantable cardioverter defibrillators (ICDs) HCM recipients [12–14]. Though patients with HOCM are traditionally viewed as having a higher symptom burden and worse clinical performance, our study did not demonstrate worse HRQoL in this patient population as compared to patients with NHOCM. As expected, patients with more severe symptoms, quantified using the NYHA functional classification system, presented a worse HRQoL with progressively lower mean KCCQ score values. PHT seems to be another important comorbidity significantly impacting global HRQoL in patients with HCM. PHT derived from TTE has been associated with increased mortality, independent of the severity of diastolic and systolic dysfunction, mitral regurgitation, symptoms, or cardiovascular comorbidities in previous studies performed in patients with either HF with preserved or reduced LVEF [15, 16]. In summary, patients with a worse HRQoL were older, presented more severe symptoms, worse renal function and echocardiographic parameters of severity and/or dysfunction like increased wall thickness, higher LV filling pressure, worse LV longitudinal function, and higher pulmonary artery systolic pressure. Kidney function and NYHA functional class emerged as important predictive factors of the HRQoL level quantified with the use of the KCCQ score in our population, explaining about 31% of the observed variance. This underscores the importance and impact of comorbidities and exercise capacity on perceived health and functionality in these patients, with implications on a focused therapeutic management of these associations. Applying the appropriate HCM therapies which are demonstrated to improve functional status (e.g. LV outflow tract obstruction reduction therapies) should therefore also ameliorate HRQoL. Indeed, improvements in distress, HRQoL and well-being have been demonstrated shortly after alcohol ablation procedures; however the preinterventional high prevalence of depression, anxiety and impaired HRQoL (reported in 57% of the patient population) represents an important clinical notice concerning the need to screen and closely evaluate these patients regarding HRQoL and derived measures [17]. More recenlty, treating HOCM patients with the oral drug mavacamten led to not only improved functional capacity and decreased LVOT gradient, but also a favorable effect on subjective well being as quantitated by the KCCQ [18, 19].

A nationwide study performed in Sweden recently demonstrates a markedly poor HRQoL in HCM patients with ICDs [14]. Although we live in an era with marked advancements in specific treatment options and overall cardiac care and comorbidities, the aforementioned study consistently showed a poor HRQoL in HCM patients with ICDs. Additionally, a similar study that included a large cohort of HCM patients with ICD implantations (486 patients) reported heightened anxiety in expectation of future shocks, but with preserved psychological well-being and HRQoL, the authors concluding that ICD therapy does not substantially impair overall psychological and physical well-being in these patients [20]. This is an important detail given the fact that recent studies report arrhythmia related deaths at a rate of about 1% per year in the HCM population [21, 22]. Little post-shock adversity with zero mortality over the first 2 years after initial ICD interventions has been found in a recent publication [20]. In our cohort, we did not find any significant difference in HRQoL between patients with and without an ICD, though we need to emphasize that our cohort included only a small number of patients with ICD. On the other hand, previous studies managed to show that HRQoL generally improves during the first year following dual-chamber pacemaker implantation for intraventricular gradient reduction [23, 24].

In the present study, significant gender differences in the global KCCQ score was observed, with a poorer global HRQoL as well as a higher symptom burden among females. This supports the concept that gender together with comorbidities may indeed determine a worse HRQoL.

### Limitations

There are several issues impacting our data related to the HRQoL in patients with HCM and HF in this exploratory study that deserve mention. First and foremost, we had a small sample size, which may have limited our power to detect certain differences between patient subgroups. We also enrolled only inpatients, which could represent a source of bias and influence our interpretation of the data. Stress echocardiography was not performed systematically in every patient, which may have misclassified some patients as non-obstructive phenotypes, but all patients underwent provocation maneuvers (mainly Valsalva). Cardiac magnetic resonance imaging data were only partially available and, as such, not used in the analysis, though certainly the refinement of data provided by this technique might have influenced our results.

## Conclusions

In conclusion, the present data extends our understanding beyond reaffirmation that patients with HCM and HF present at least a moderate degree of alteration in quality of life. This is especially true for patients with pulmonary hypertension and more severe functional impairment. Renal failure and NYHA class are potential markers of quality of life in clinical practice.

## Data Availability

Data is available upon reasonable request sent to the corresponding author.

## Abbreviations

BNP: Brain natriuretic peptide
EACVI: European Association of Cardiovascular Imaging
eGFR: Estimated glomerular filtration rate
HCM: Hypertrophic cardiomyopathy
HF: Heart failure
HOCM: Obstructive hypertrophic cardiomyopathy
HRQoL: Health related quality of life
ICD: Implantable cardioverter defibrillator
IVS: Interventricular septum
KCCQ: Kansas City Cardiomyopathy Questionnaire
LA: Left atrium
LVEF: Left ventricular ejection fraction
LVESD: Left ventricular end-systolic dimension
LVH: Left ventricular hypertrophy
MDRD: Modification of Diet in Renal Disease equation
NHOCM: Non-obstructive hypertrophic cardiomyopathy
NYHA: New York Heart Association
PASP: Pulmonary artery systolic pressure
PAT: Pulmonary acceleration time
PHT: Pulmonary hypertension
PW: Posterior wall
TTE: Transthoracic echocardiography
